# Adipocytokine Levels in Genetically High Risk for Type 2 Diabetes in the Indian Population: A Cross-Sectional Study

**DOI:** 10.1155/2012/386524

**Published:** 2012-11-14

**Authors:** K. Subhash Chandra Bose, Shachin K. Gupta, Prerna Vyas

**Affiliations:** ^1^Department of Biochemistry, L.N. Medical College, Kolar Road, Bema Kunj, Bhopal 462042, India; ^2^Department of Medicine, L.N. Medical College and Research Centre, Kolar Road, Bhopal, Madhya Pradesh, India

## Abstract

*Introduction*. In view of the noteworthy role of adipocytokines in the onset of insulin resistance and diabetes in gene-knockout-rat-model-cell-line studies we aimed to study the influence of genetic predisposition for diabetes on adipocytokine levels and their role in building insulin-resistance-like environment well before the onset of diabetes; thus a hypothesis can be drawn on their role in developing diabetes in high risk population. *Methods*. Ages between 18 and 22 years were selected and divided into three groups. Group I (*n* = 81): control group with no family history of diabetes. Group II (*n* = 157): with one of their parents with history of type 2 diabetes. Group III (*n* = 47): with both parents having history of type 2 diabetes. In all the groups we estimated fasting plasma glucose, insulin and adipocytokines like adiponectin, leptin, TNF-**α**, and IL-6. *Results*. Of all adipocytokines we observed significantly lower levels of adiponectin (8.7 ± 1 **μ**g/mL in group III and 9.5 ± 1.3 **μ**g/mL group II) when compared to control (11.0 ± 1.2 **μ**g/mL; *P* < 0.01) and it has strong correlation with family history of diabetes with Pearson's coefficient of −0.502. Linear regression analysis showed significant negative association with HOMA-IR (*P* < 0.01) and logistic regression analysis showed highest association with parental diabetes (*P* < 0.01; OR .260, 95% CI .260–.468). *Conclusion*. Genetic predisposition for diabetes may influence adiponectin gene expression leading to decrease in its plasma concentration, which might play a key role in developing diabetes in near future.

## 1. Introduction

 As per International Diabetic Federation, India is so called as diabetic capital of the World with already 50.8 million existing diabetic patients, expected to rise up to 87 million by 2030 [1]. In early 90s initially by Ramaiya et al. [2] and later by Chandalia et al. [3] their studies identified the ethnic susceptibility of Indians to type 2 diabetes, insulin resistance (IR) and cardiovascular diseases (CVD) compared to other ethnic groups. Later on many Indian studies have revealed that though prevalence of obesity is low, Indians tend to have increased waist circumference, that is, central obesity and high fat %, when compared to other ethnic groups with same BMI. This is main attributing factor for increased visceral fat [4, 5]. In recent times this unique feature hard-pressed the scientific community to coin the term *Asian Indian Phenotype*. 

 In recent times it has been observed that adipose tissue is not just an energy storage organ but it is an active endocrine organ secreting many cytokines like leptin, adiponectin, TNF-*α*, IL-6, and RBP-4, collectively termed as adipocytokines. Though the contributory role of these adipocytokines in insulin resistance, diabetes, and CVD have been recognized [6–8], no studies are available on which one of these adipocytokines triggers first in the development of IR and diabetes. Many studies were done to date on the altered levels of these adipocytokines either in well diagnosed diabetics or during the antidiabetic drug therapy, but no studies were done on the influence of genetic predisposition on plasma levels and their prospective role in initiation and propagation of IR and diabetes in humans. Thus we aimed this study to assess the serum levels of these adipocytokines in genetically high risk for diabetes population whose parents are type 2 diabetic, hence insulin resistance and diabetes triggering adipocytokine can be identified, so that a different pharmacological and therapeutic approach can be made in treating type 2 diabetes.

## 2. Methods and Materials

 This cross-sectional study is approved by institutional ethics committee with wide Ref No: IEC/3/2011 and written consent was taken from all participants. For this study we selected the participants and grouped in three groups based on their family history of type 2 diabetes. All the participants enrolled for our study were students of our flagship institutions comprising Medical, Dental, and Nursing sciences. All are on vegetarian diet with identical ingredients as they are sharing same kitchen and are residing in one campus.

**Group I**

Control group (*n* = 81) consisting of both males and females of age group between 18 to 22 years, irrespective of BMI whose parents are nondiabetic, nonhypertensive, and do not have any family history of coronary heart diseases.

**Group II**

Both males and females (*n* = 157) of age group between 18 to 22 years, irrespective of BMI with one of their parents with history of type 2 diabetes.

**Group III**

Both males and females (*n* = 47) of age group between 18 to 22 years, irrespective of BMI, with both parents having history of type 2 diabetes.

**Exclusion Criteria**

None of the subjects from above groups is diabetic, pregnant, has gestational diabetic, and none of their parents was type 1 diabetic. The diabetes in these subjects was excluded through applying WHO criteria [9].

**Measurements**

All the subjects of above groups are on overnight fasting. In all the above subjects we measured serum glucose and Hba1c by commercial kits by Biosystems A25 fully auto analyzer. Serum insulin and adipocytokines like leptin, adiponectin, IL-6, and TNF-*α* were estimated by ELISA method with commercial kits. Blood pressure was measured by Omron HEM-7203 automated BP monitor instrument, and average of three measurements was taken into consideration.

Insulin resistance was measured by homeostasis model for assessment (HOMA) based on [10]:

(1) HOMA-IR=fasting  serum  insulin  (μIU/mL)×fasting  serum  glucose  (mg/dL)/405.

### 2.1. Statistical Analysis

Descriptive results of continuous variables are expressed as mean ± SD for normally distributed or as median for nonparametrically distributed variables. Comparison between study groups and control was done by one-way ANOVA, Student *t*-test, or Mann Whitney *U* test whichever is appropriate. Relationship between continuous variables was expressed by applying Pearson's correlation (*r*) for normally distributed variables and Spearman's correlation for nonparametric distribution. *P*  value < 0.05 is considered significant and <0.01 as highly significant.

 Multivariate linear regression was performed in stepwise manner to evaluate the association among independent and dependent variables. Multivariate logistic regression analysis was performed to evaluate the association of adipocytokines as independent variables with parental diabetes as dependent variable in a separate model after adjusting for potential cofounders. *P*  value < 0.05 considered to be statistically significant and *P* < 0.01 as highly significant. All the data were analyzed using statistical software SPSS version 19.

## 3. Results

### 3.1. Plasma Adipocytokines

In the present study (Table 1) we observed statistically highly significant lower levels of adiponectin 8.7 ± 1 in study population whose both parents are diabetic and 9.5 ± 1.3 in population of single parent diabetic when compared to control 11.0 ± 1.2 who do not had any family history of diabetes (*P* < 0.01). Even the mean difference of adiponectin levels between single parent diabetic and both parents diabetic was significant *P* < 0.01 (Table 2). We in this study observed no statistically difference in the mean levels of TNF-*α*, IL-6, and leptin between genetically high risk for diabetes population and control group *P* > 0.5 (Table 1). At the same time the same no significant difference in serum levels of these adipocytokines between single and both parents diabetic groups *P* > 0.05 was observed (Table 2).

### 3.2. Plasma Insulin and Other Diabetic Markers

From Table 1 we observed very high levels of plasma insulin, glucose, and HOMA-IR levels in genetically high risk population than the control group *P* < 0.01. As the participants of all the three groups contain obese and overweight, though significant not highly significant variation of BMI between groups was observed in our study *P* < 0.05. No statistical difference in WHR between groups was observed (*P* > 0.05) (Table 1). As Table 2 shows no statistically difference in mean values of these variables between single parent diabetic and both parents diabetic groups (*P* > 0.05) was observed.

### 3.3. Correlation and Association of Plasma Adipocytokines and BMI with Genetically Predisposition for the Development of Diabetes

In examining the relationship between plasma adipocytokines with parental diabetes, we observed that adiponectin has highest correlation with Pearson's coefficient (−0.502 and *P* < 0.01) and no correlation with other adipocytokines like TNF-*α*, IL-6, and leptin; *P* > 0.05 (Table 3).

In assessing the association of adipocytokines with parental diabetes as dependent variable in feature onset of diabetes in genetically high risk population, we observed statistically highest association of adiponectin with parental diabetes *β*  −.675 and *P* < 0.01 in comparison with other adipocytokines (Table 4). In annoying to assess the association of adipocytokines with the development of insulin resistance (HOMA-IR) among the genetically high risk population adiponectin showed highest association with *β*  −.400 and *P* < 0.01 (Table 5). 

From Table 6, multivariate logistic regression in stepwise manner analysis with parental diabetes as dependent variable, we observed that adiponectin has highest degree of association in developing diabetes and insulin resistance in genetically high risk population even after being adjusted for other confounding factors (OR 0.39 with *P* < 0.01).

## 4. Discussion

In this study we assessed the change in blood glucose levels and insulin resistance (IR) status well before the onset of diabetes in population with high degree of positive family history for diabetes and tried to understand the possible role and influence of adipocytokines in this population in initiating the lead towards developing diabetes in near future. 

 Of baseline adipocytokine levels between control and study groups ironically we observed statistically highly significant lower levels of plasma adiponectin in population whose both parents were diabetic (8.7 ± 1.0 *μ*g/mL and 9.5 ± 1.3 *μ*g/mL) in single parent diabetic group when compared to control (11.0 ± 1.2 (Table 1) *P* < 0.01). We observed no difference in mean levels of other adipocytokines like leptin, IL-6 and TNF-*α* with *P* > 0.05 (Table 1).

As in Table 3 shows, of all the adipocytokines adiponectin only shows highest correlation with Pearson's coefficient −0.502 (*P* < 0.01) indicating genetic predisposition for diabetes which initially leads to abnormal adiponectin synthesis, thus kick off IR, leading to development of diabetes. From multivariate stepwise linear regression (Table 4) of all adipocytokines only adiponectin has highest association (beta coefficient −0.675 and *P* < 0.01) with parental diabetes as dependent variable. This may be due to some genetic factors that might influence the expression of adiponectin gene in high risk population.

In Multivariate logistic regression analysis with parental diabetes as dependent variable (Table 6), adiponectin showed highest association with OR 0.349, 95% CI 0.260–0.468, and *P* < 0.01. Even after adjusted for compounding factors like TNF-*α*, IL-6, and leptin, adiponectin stood as independent marker of association with genetic predisposition for future development of diabetes (*P* < 0.01).

These results have potential implications in evolving the hypothesis of who triggers first in developing diabetes in genetically high risk population. The novel observation of significant impact of adiponectin on IR and blood glucose homeostasis in genetically high risk for diabetes population may have clinical relevance. As per Kissebah et al. [11] the human adiponectin gene has been localized to chromosome 3p27 which has susceptibility locus for the metabolic syndrome, which could suggest an influence of abnormal synthesis of adiponectin in initiation or perpetuation of metabolic syndrome in genetically high risk population. Study done by Lihn et al. [12] observed negative correlation of adiponectin levels with visceral adiposity and lower gene expression in visceral fat compared to subcutaneous fat in both lean and obese humans. Genetic high risk population in our study may have higher visceral than subcutaneous fat, which might have led to decreased adiponectin levels. Irrespective of BMI we observed overall decrease in adiponectin levels in study group that were highly correlated with IR. The study done by Weyer et al. [13] also showed circulating adiponectin levels much correlated with hyperinsulinemia and IR than with obesity or body fat. The same was observed in our study as there is no statistical difference in WHR between control and study groups but adiponectin levels are far lower in study population. The mean difference in blood glucose levels between control and study groups may be because of suppression of hepatic gluconeogenesis due to decreased adiponectin levels and the same effect of adiponectin infusion on hepatic gluconeogenesis was observed by Combs et al. [14]. The hypoglycemic effect of adiponectin was also supported by Berg et al. [15] in their study on administration of recombinant adiponectin they observed reduction in serum glucose in normal and diabetic rodents without stimulating insulin secretion. In a clinical trial done by Gerstein and his associates [16] they observed that Rosiglitazone reduces the risk of progression to diabetes in subjects who had higher baseline BMI by enhancing both *β* cell function and adiponectin synthesis. On Troglitazone treatment the same increase in adiponectin mRNA expression in adipose tissue of both obese and lean rodents was observed by Maeda et al. [17] in their experimental study on rodents. By the study done by Yu and his team [18] on 3 month Troglitazone treatment it reversed the inhibitory effect of TNF-*α* on expression of adiponectin thus reversing glucose intolerance in obese or lean subjects. From multivariate linear regression analysis with adipocytokines and BMI as independent variables and HOMA-IR as dependent variable (Table 5) we observed HOMA-IR strongly associates with adiponectin (standard coefficient beta  −.4 and *P* < 0.01) than with BMI (standard coefficient beta  .287 and *P* > 0.05) indicating prospective role of adiponectin in insulin sensitizing activity. In accordance to our study, in an experimental study done by Fu et al. [19] they observed adiponectin overexpression increased insulin's ability to maximally stimulate glucose uptake by 78% through increased GLUT-4 gene expression.

From many studies [20, 21] at high levels of TNF-*α* and IL-6 the inhibitory effect on expression of adiponectin gene expression was observed, thus decreasing the levels of plasma adiponectin. But in our study we observed more much normal levels of these adipocytokines in study group as like in controls indicating well prior to adverse effect of TNF-*α* and IL-6 on adiponectin gene expression, there must be other factors influencing its expression in genetically high risk population, thus leading to early creation of IR-like environment ultimately leading to development of diabetes.

## 5. Conclusion

 From this study, of all adipocytokines, adiponectin levels were grossly reduced in genetically high risk for diabetes population, indicating the influence of genetic factors in expressing adiponectin gene and from linear and logistic regression analysis adiponectin has highest association with IR indicating its probable role in future onset of diabetes in this population.

## Figures and Tables

**Table 1:** Showing baseline anthropometric and biochemical characteristics of three study groups.

Parameters	Non-diabetic (*n* = 81)	Single parent diabetic (*n* = 157)	Both parents diabetic (*n* = 47)	*P* value
BMI (kg/m^2^)	21.8 ± 3.3	22.4 ± 4.1	23.6 ± 3.5	0.042*
WHR	0.81 (0.79–0.83)	0.82 (0.80–86)	0.82 (0.83–88)	0.184^#^
BP—systolic (mmHg)	110.46 ± 5.9	110.29 ± 6.7	110.49 ± 4.9	0.996^#^
BP—diastolic (mmHg)	75.40 ± 3.9	74.43 ± 3.7	75.68 ± 4.1	0.083^#^
Fasting serum insulin (*μ* IU/mL)	8.06 ± 0.99	10.79 ± 1.5	11.12 ± 1.3	<0.001**
Fasting serum glucose (mg/dL)	73.4 ± 8.5	78.02 ± 10.1	82.4 ± 9.7	<0.001**
2 h glucose of OGTT (mg/dL)	115.0 ± 6.4	116.9 ± 5.5	117.0 ± 5.9	0.060^#^
HbA1c (%)	4.3 ± 0.55	4.5 ± 0.49	4.53 ± 0.510	0.298^#^
HOMA-IR	1.45 ± 0.23	2.09 ± 0.49	2.26 ± 0.41	<0.001**
TNF-*α* (pg/mL)	15.24 ± 3.5	16.06 ± 5.1	17.55 ± 5.8	0.038*
IL-6 (pg/mL)	112.3 ± 19.4	114.3 ± 19.9	119.0 ± 18.8	0.176^#^
Leptin (ng/mL)	12.3 ± 3.3	12.7 ± 1.8	13.1 ± 1.7	0.199^#^
Adiponectin (*μ*g/mL)	11.0 ± 1.2	9.5 ± 1.3	8.7 ± 1.0	<0.001**

Results are presented as mean ± SD or median (interquartile range, 25–75%). **P < 0.01; *P < 0.05; ^#^ *P* > 0.05; BMI: body mass index; WHR: waist to hip ratio; BP: blood pressure; OGTT: oral glucose tolerance test; HbA1c: glycated hemoglobin; HOMA-IR: homeostasis model assessment-insulin resistance; TNF-*α*: tumor necrotic factor-alpha; IL-6: interleukin-6.

**Table 2:** Showing mean difference in serum RBP-4 and other biomarkers between single parent diabetic and both parents' diabetic groups.

Parameters	Single parent diabetic group (*n* = 147)	Both parents diabetic group (*n* = 47)	*P* value
BMI	22.48 + 4.1	23.63 + 3.5	0.086^#^
WHR	0.82 (0.80–0.86)	0.82 (0.83–0.88)	>0.05^#^
Systolic BP	110.29 ± 6.7	110.49 ± 4.9	0.879^#^
diastolic BP	74.43 ± 3.7	75.68 ± 4.1	0.074^#^
Fasting serum insulin	10.79 ± 1.5	11.12 ± 1.3	0.192^#^
Fasting serum glucose	78.0 ± 10.9	82.44 + 9.7	0.010**
2 h glucose of OGTT	116.9 ± 5.5	117.0 ± 5.9	0.934^#^
HbA1c	4.5 ± 0.49	4.53 ± 0.510	0.136^#^
HOMA-IR	2.09 + 0.49	2.26 ± 0.41	0.030*
TNF-*α* (pg/mL)	16.06 ± 5.1	17.55 ± 5.8	>0.05^#^
IL-6 (pg/mL)	114.3 ± 19.9	119.0 ± 18.8	>0.05^#^
Leptin (ng/mL)	12.7 ± 1.8	13.1 ± 1.7	>0.05^#^
Adiponectin (*μ*g/mL)	9.5 ± 1.3	8.7 ± 1.0	<0.01**

Results are presented as mean + SD, or median (interquartile range, 25–75%). **P < 0.01; *P < 0.05; ^#^ *P* > 0.05; BMI: body mass index; WHR: waist to hip ratio; BP: blood pressure; OGTT: oral glucose tolerance test; HbA1c: glycated hemoglobin; HOMA-IR: homeostasis model assessment-insulin resistance; TNF-*α*: tumor necrotic factor-alpha; IL-6: interleukin-6.

**Table 3:** Showing correlation between parental diabetes and biological parameters in study groups.

Parameters	Pearson's coefficient	*P* value
BMI (kg/m^2^)	0.107	0.075^#^
Fasting serum insulin (*μ*IU/mL)	0.686	0.000**
Fasting serum glucose (mg/dL)	0.257	0.000**
HOMA-IR	0.592	0.000**
TNF-*α* (pg/mL)	0.109	0.071^#^
IL-6 (pg/mL)	0.072	0.231^#^
Leptin (ng/mL)	0.087	0.150^#^
Adiponectin (*μ*g/mL)	−0.502	0.000**

**P < 0.01; ^#^ *P* > 0.5; BMI: Body Mass Index; HOMA-IR: homeostasis model assessment-insulin resistance; TNF-*α*: Tumor Necrotic Factor-alpha; IL-6: Interleukin 6.

**Table 4:** Showing multivariate linear regression in stepwise manner for the relationship between adipocytokines and BMI with parental diabetes as dependent variable.

Model	Unstandardized coefficient		Standardized coefficient	
	B	Std. Error	Beta	*P* value
1 (constant)	3.554	0.329		0.000**
BMI (kg/m^2^)	−0.027	0.017	−0.226	0.116^#^
Adiponectin (*μ*g/mL)	−0.206	0.019	−0.675	0.000**
Leptin (ng/mL)	0.012	0.015	0.061	0.440^#^
IL-6 (pg/mL)	−0.003	0.002	−0.126	0.138^#^
TNF-*α* (pg/mL)	−0.001	0.009	−0.009	0.933^#^

**P < 0.01; ^#^ *P* > 0.5; BMI: body mass index; TNF-*α*: tumor necrotic factor-alpha; IL-6: interleukin-6.

**Table 5:** Showing multivariate linear regression in stepwise manner for the relationship between adipocytokines and BMI with HOMA-IR as dependent variable.

Model	Unstandardized coefficient		Standardized coefficient	
	B	Std. Error	Beta	*P* value
1 (constant)	2.894	0.383		0.000**
BMI (kg/m^2^)	0.039	0.020	0.287	0.051^#^
Adiponectin (*μ*g/mL)	−0.140	0.022	−0.400	0.000**
Leptin (ng/mL)	−0.030	0.18	−1.37	0.089^#^
IL-6 (pg/mL)	−0.001	0.002	−0.042	0.626^#^
TNF-*α* (pg/mL)	0.003	0.011	−0.030	0.770^#^

**P < 0.01; ^#^ *P* > 0.5; BMI: body mass index; TNF-*α*: tumor necrotic factor-alpha; IL-6: interleukin-6.

**Table 6:** Showing multivariate logistic regression in stepwise manner to assess the association of adiponectin and other adipocytokines with parental diabetes.

Parameter	Odds ratio (OR)	95% confidence interval CI	*P* value
Adiponectin	0.349	0.260–0.468	0.000**
Unadjusted			
Adjusted for leptin	0.299	0.216–0.416	0.000**
Adjusted for TNF-*α*	0.282	0.200–0.397	0.000**
Adjusted for IL-6	0.274	0.190–0.386	0.000**

**P < 0.01; TNF-*α*: tumor necrotic factor-alpha; IL-6: interleukin-6.
